# Association between Glutathione S-Transferase T1 Null Genotype and Gastric Cancer Risk: A Meta-Analysis of 48 Studies

**DOI:** 10.1371/journal.pone.0060833

**Published:** 2013-04-09

**Authors:** Weiyuan Ma, Le Zhuang, Bo Han, Bo Tang

**Affiliations:** 1 Department of Dermatology, Qilu Hospital, Shandong University, Jinan, China; 2 Institute of Pathology, School of Medicine, Shandong University, Jinan, China; 3 Department of Oncology, Southwest Hospital, the Third Military Medical University, Chongqing, China; Pontificia Universidad Catolica de Chile, Chile

## Abstract

**Background:**

Glutathione S-transferases (GSTs) have proved to be involved in the detoxifying several carcinogens and may play an important role in carcinogenesis of cancer. Previous studies on the association between Glutathione S-transferase T1 (*GSTT1*) polymorphism and gastric cancer risk reported inconclusive results. To clarify the possible association, we conducted a meta-analysis of eligible studies.

**Methods:**

We searched in the Pubmed, Embase, and Wangfang Medicine databases for studies assessing the association between *GSTT1* null genotype and gastric cancer risk. The pooled odds ratio (OR) and its 95% confidence interval (95%CI) was calculated to assess the strength of the association. A total of 48 studies with a total of 24,440 individuals were ultimately eligible for meta-analysis.

**Results:**

Overall, *GSTT1* null genotype was significantly associated with increased risk of gastric cancer (Random-effect OR = 1.23, 95%CI 1.13–1.35, P _OR_ <0.001, I^2^ = 45.5%). Significant association was also found in Caucasians, East Asians, and Indians (P _Caucasians_ = 0.010; P _East Asians_ = 0.003; P _Indians_ = 0.017). After adjusting for other confounding variables, G*STT1* null genotype was also significantly associated with increased risk of gastric cancer (Random-effect OR = 1.43, 95%CI 1.20–1.71, P _OR_ <0.001, I^2^ = 48.1%).

**Conclusion:**

The meta-analysis provides strong evidence for the significant association between *GSTT1* null genotype and increased risk of gastric cancer.

## Introduction

Gastric cancer is the second most frequent cause of cancer death worldwide, and the global burden of gastric cancer continues to increase largely in economically developing countries [1], [2]. Though there are many achievements in the treatment of gastric cancer in terms of the combined therapy, novel anti-tumor agents and personalized treatments the, the survival of gastric cancer patients is still poor [3], [4]. Currently, the prevention intervention is regarded as the best option to reduce the high rated of gastric cancer mortality. Effective prevention strategies should be based on specific risk profiles of gastric cancer, including Helicobacter pylori, environmental factors, and the host genetic polymorphisms [2]. In addition, genetic susceptibility to gastric cancer has been a research focus, and identifications of risk factors for gastric cancer are important for us to understand the biology of gastric carcinogenesis and develop some effective interventions. Glutathione S-transferases (GSTs) have proved to be involved in the detoxifying several carcinogens and may play an important role in carcinogenesis of cancer [5]–[7]. The theta class of GSTs is encoded by the Glutathione S-transferase T1 (*GSTT1*) gene located on the long arm of chromosome 22 (22q11.23), and the homozygous deletion (null genotype) of *GSTT1* gene causes complete absence of GST enzymes activity [8]. Previous studies on the association between Glutathione S-transferase T1 (GSTT1) polymorphism and gastric cancer risk reported inconclusive results [9]–[48]. To clarify the possible association, we conducted a meta-analysis of eligible studies by searching three electronic databases.

## Methods

### Identification of Eligible Studies

We searched in the Pubmed, Embase, and Wangfang Medicine databases for studies assessing the association between *GSTT1* null genotype and gastric cancer risk. The literature strategy used the following keywords: (‘‘Glutathione S-transferase T1’’, ‘‘*GSTT1*’’ or ‘‘*GSTT*’’) and (‘‘gastric cancer’’, ‘‘gastric carcinoma’’, ‘‘stomach cancer’’ or ‘‘stomach carcinoma’’). The references of the retrieved articles were also hand searched at the same time to identify additional published articles. The references of eligible studies and relevant reviews were also checked for other literature not indexed into common databases. There was no language restriction applied in this meta-analysis. The inclusion criteria of eligible studies were as following: (1) Case-control study; (2) The cases were patients with histopathologicaly proved gastric cancer; (3) The controls were gastric cancer-free individuals; (4) Reported the frequencies of GSTT1 polymorphism in both cases and controls or the odds ratio (OR) and its 95% confidence interval (95%CI) of the association between *GSTT1* null genotype and gastric cancer risk. Family-based studies and studies containing overlapping data were all excluded.

### Data Extraction

Relevant data were extracted from all the eligible studies independently by two reviewers, and disagreements were settled by discussion and the consensus among all reviewers. The main data extracted from the eligible studies were as following: the first author, year of publication, country, ethnicity, characteristics of cases, characteristics of controls, total numbers of cases and controls, the genotype frequency of GSTT1 polymorphism, adjusted variables, and adjusted ORs and corresponding 95%CIs. Different ethnicities were mainly categorized as Caucasians, East Asians, Indians, Africans, and Mixed. If a study did not specify the ethnicity or if it was not possible to separate participants according to such phenotype, the group was termed ‘‘mixed’’. For studies including subjects of different ethnic populations, data were collected separately whenever possible and recognized as an independent study.

### Quality Assessment

Quality of eligible studies in present meta-analysis was assessed using the Newcastle Ottawa scale (NOS) as recommended by the Cochrane Non-Randomized Studies Methods Working Group. This instrument was developed to assess the quality of nonrandomized studies, specifically cohort and case-control studies [49]. This scale awards a maximum of nine stars to each study: four stars for the adequate selection of cases and controls, two stars for comparability of cases and controls on the basis of the design and analysis, and three stars for the adequate ascertainment of the exposure in both the case and control groups. Given the variability in quality of eligible studies found on our initial literature search, we considered studies that met 5 or more of the NOS criteria as high quality.

### Statistical Methods

The strength of the association between *GSTT1* null genotype and gastric cancer risk was assessed by calculating the pooled OR with its corresponding 95%CI, and the significance of the pooled OR was determined by the Z-test. To assess the heterogeneity among the included studies more precisely, both the chi-square based Q statistic test (Cochran's Q statistic) to test for heterogeneity and the I^2^ statistic to quantify the proportion of the total variation due to heterogeneity were calculated [50], [51]. If obvious heterogeneity existed among those included studies (P _Q statistic_ <0.05), the random-effect model (DerSimonian and Laird method) was used to pool the results [52]. When there was no obvious heterogeneity existed among those included studies (P _Q statistic_ >0.05), the fixed-effect model (Mantel-Haenszel’s method) was used to pool the results [53]. Subgroup analyses were performed by ethnicity, the adjusted status of the estimates, and the quality of studies. The kinds of ethnicity were mainly defined as Caucasians, East Asians, and Indians. Publication bias was investigated with the funnel plot and its asymmetry suggested risk of publication bias. The asymmetry of funnel plots was further assessed by both the Begg’s test and the Egger’s linear regression test [54], [55]. All statistical tests for this meta-analysis were performed with STATA (version 11.0; Stata Corporation, College Station, TX). A P value less than 0.05 was considered statistically significant, and all the P values were two sided.

## Results

### Studies Selection and Characteristics of Eligible Studies

There were 107 relevant abstracts identified by the searching words, and 48 studies were firstly excluded after the careful review of the abstracts, leaving 59 studies for full publication review (Figure S1). Of those 59 studies, 11 studies were excluded (5 for containing overlapping data, 2 for reviews, 2 for without adequate data, and 2 for on GSTM1 polymorphism). Therefore, a total of 48 studies with a total of 24,440 individuals were ultimately eligible for meta-analysis [9]–[48], [56]–[63]. The main characteristics of those 48 studies were presented in Table 1 (Table 1). There were 25 studies from East Asians [12], [13], [16], [19]–[21], [23], [27]–[30], [33], [34], [37], [38], [44], [48], [56]–[63], 16 ones from Caucasians [9]–[11], [14], [15], [17], [22], [25], [26], [31], [32], [35], [36], [39], [43], [47], 5 from Indians [24], [41], [42], [45], [46], and the left two from the others populations [18], [40]. There were 18 studies reporting the adjusted ORs, and 5 reporting the ORs adjusted for H. pylori infection (Table 1). Multiplex-polymerase chain reaction (Multiplex-PCR) was the most common genotype method of GSTT1 polymorphism (Table 1). According to the NOS scale, there were 43 studies with high quality, and 5 with low quality (Table 1).

**Table 1:** Characteristics of 48 eligible studies in this meta-analysis.

First author(Year)	Country	Ethnic group	Cases	Controls	Adjusted potential confounding variables	Genotype Methods*	Quality assessment
Jing (2012) [20]	China	East Asians	410 newly diagnosed and histological confirmed gastric cancer cases	410 controls which consisted of participants in the health examination center and were matched with the cases by age and sex	Adjusted for sex, age, drinking, smoking and H. pylori infection	PCR-CTPP	7
Garcia-Gonzalez (2012) [17]	Spain	Caucasians	711 consecutive Spanish Caucasian patients with newly diagnosed gastric cancer	557 Spanish, Caucasian, cancer-free volunteers with no previous history of gastric disease, matched by gender, age and area of residence.	None.	Multiplex PCR	7
Tripathi (2011) [42]	India	Indians	88 patients with pathologically confirmed gastric cancer	89 healthy volunteers from community	None.	Multiplex PCR	4
Yadav (2011) [45]	India	Indians	41 patients with pathologically confirmed gastric cancer	130 healthy geographically and racially matched controls	None.	Multiplex PCR	5
Zhang (2011) [48]	China	East Asians	194 histological confirmed gastric cancer cases	436 controls recruited from health individuals visiting hospital for routine physical examination	Adjusted for sex, age, drinking, smoking, family cancer history and H. pylori infection.	PCR-CTPP	6
Luo (2011) [23]	China	East Asians	123 pathologically confirmed patients with gastric cancer	129 healthy controls who are cancer- and hematological disease-free.	None.	Multiplex PCR	5
Li (2011) [60]	China	East Asians	585 newly diagnosed and histological confirmed gastric cancer cases	585 non-cancer controls	None.	Multiplex PCR	6
Yadav (2010) [46]	India	Indians	133 histologically confirmed cases with gastric cancer	270 unrelated voluntary healthy individuals who were accompanying the patients to the hospital	Adjusted with all other risk variables under consideration.	Multiplex PCR	6
Palli (2010) [31]	Italy	Caucasians	314 histologically confirmed gastric cancer patients	548 healthy controls	None.	Multiplex PCR	6
Nguyen (2010) [30]	Vietnam	East Asians	59 histological confirmed gastric cancer cases	100 non-cancer patients	Adjusted forage, gender, current drinking, and current smoking.	Multiplex PCR	5
Piao (2009) [33]	Korea	East Asians	2213 newly diagnosed gastric cancer cases	1699 participants in the Thyroid Disease Prevalence Study	None.	TaqMan assays	7
Zendehdel (2009) [47]	Sweden	Caucasians	124 newly diagnosed native Swedish patients with gastric cancer	470 cancer-free native Swedes matched on age and sex distribution	Adjusted for sex and age	Multiplex PCR	7
Moy (2009) [27]	China	East Asians	312 incident gastric cancer cases	735 controls matched to the index case by date of birth, date of biospecimen collection and neighborhood of residence at recruitment	None.	TaqMan assays	6
Malik (2009) [24]	India	Indians	108 untreated histologically confirmed cases with gastric cancer	195 untreated and healthy controls	Adjusted for gender and age	Multiplex PCR	7
Al-Moundhri (2009) [10]	Oman	Caucasians	107 unrelated gastric cancer patients	107 non-cancer patients attending outpatient clinics, and blood donors.	Adjusted for sex and age	Multiplex PCR	7
Masoudi (2009) [26]	Iran	Caucasians	92 histologically confirmed cases with gastric cancer	134 sex and age frequency-matched controls randomly selected from the healthy blood donors.	Adjusted for other risk factors.	Multiplex PCR	7
Tripathi (2008) [41]	India	Indians	76 unrelated gastric cancer patients	100 untreated and healthy controls	None.	Multiplex PCR	4
Xie (2008) [62]	China	East Asians	70 newly diagnosed and histological confirmed gastric cancer cases	100 controls were recruited from health individuals visiting hospital for routine physical examination.	None.	Multiplex PCR	5
Boccia (2007) [11]	Italy	Caucasians	105 consecutive primary gastric cancer patients	254 cancer-free patients frequency matched to cases for age (±5 years) and gender	Adjusted for age and gender	Multiplex PCR	7
Wideroff (2007) [43]	USA	Caucasians	105 consecutive gastric cancer patients	208 controls frequency matched to expected age and sex distributions	None.	Multiplex PCR	4
Ruzzo (2007) [35]	Italy	Caucasians	126 H. pylori-negative patients with sporadic diffuse gastric cancer	144 healthy adult donors with no family history of diffuse gastric cancer	Adjusted for age and sex.	Multiplex PCR	6
Agudo (2006) [9]	10 European countries	Caucasians	242 cases with newly diagnosed gastric cancer	932 control subjects matched by center, gender, age, and date of blood collection	Adjusted for sex, age, center, and date of blood extraction.	Multiplex PCR	8
Martinez (2006) [25]	Spain	Caucasians	87 histologically confirmed cases with gastric cancer	329 unrelated and healthy individuals were included as controls.	None.	Multiplex PCR	5
Hong (2006) [19]	Korea	East Asians	108 histologically confirmed cases with gastric cancer	238 healthy subjects	None.	Multiplex PCR	6
Nan (2005) [29]	Korea	East Asians	421 gastric cancer patients	632 age- and sex-matched controls.	None.	Multiplex PCR	5
Mu (2005) [28]	China	East Asians	206 newly diagnosed cases with gastric cancer	415 healthy control subjects	Adjusted for age, gender, education, income, H. pylori infection, stomach disease history and others	Multiplex PCR	8
Palli (2005) [32]	Italy	Caucasians	175 histologically confirmed gastric cancer patients	546 healthy controls randomly sampled from the general population of Tuscany	Adjusted for age, sex, area of residence, H. pylori seropositivity and each genotype	Multiplex PCR	8
Tamer (2005) [39]	Turkey	Caucasians	70 patients with gastric cancer diagnosed by operation and histological confirmation	204 control subjects selected among healthy persons	Adjusted for sex, gender, and smoking.	Real-time PCR	7
Shen (2005) [59]	China	East Asians	121 histologically confirmed cases with gastric cancer	121 sex- and age-matched controls	None.	Multiplex PCR	5
Roth (2004) [34]	China	East Asians	90 cases of gastric cancer	454 non-cancer patients	None.	Real-time PCR	5
Gonzalez (2004) [18]	Costa Rica	Others	31 with gastric cancer	51 normal controls confirmed by X-rays (double-contrast) or endoscopic diagnosti	None.	Multiplex PCR	4
Torres (2004) [40]	Colombia	Others	46 with gastric cancer	96bnormal controls	None.	Multiplex PCR	4
Colombo (2004) [14]	Brazil	Caucasians	100 patients with histologically confirmed diagnosis of gastric cancer	150 healthy volunteers with no previous history of gastric disease, matched to the patients with respect to age, gender and ethnicity.	None.	Multiplex PCR	5
Shen (2004) [61]	China	East Asians	60 histologically confirmed cases with gastric cancer	60 sex- and age- matched controls	Adjusted for sex, gender, drinking, and smoking.	Multiplex PCR	6
Choi (2003) [13]	Korea	East Asians	80 patients with curatively resected cancer and pathologically confirmed diagnosis of gastric cancer	177 healthy cancer-free individuals	None.	Multiplex PCR	5
Qian (2003) [63]	China	East Asians	90 patients with gastric cancer	90 sex- and age-matched controls	None.	Multiplex PCR	6
Ye (2003) [57]	China	East Asians	56 patients with histologically confirmed gastric cancer	56 healthy controls	None.	Multiplex PCR	5
Gao (2002) [16]	China	East Asians	153 cases of stomach cancer	223 population-based controls	Age and sex-adjusted	Multiplex PCR	7
Wu (2002) [44]	Taiwan	East Asians	356 cases with histologically diagnosed gastric cancer	278 unaffected controls.	None.	Multiplex PCR	6
Zheng (2002) [58]	China	East Asians	92 patients with gastric cancer	92 healthy individuals	None.	Multiplex PCR	5
Shen (2002) [56]	China	East Asians	112 patients with histologically confirmed gastric cancer	662 healthy cancer-free individuals	None.	Multiplex PCR	5
Cai (2001) [12]	China	East Asians	95 incidence gastric cancer cases	104 controls selected from the same geographical region, and matched to cases by their gender and age	None.	Multiplex PCR	5
Lan (2001) [22]	Poland	Caucasians	293 patients with newly diagnosed with gastric cancer	418 controls with frequency-matched to cases by gender and by age in 5-year strata	Adjusted for age, gender, education, tobacco smoke, years lived on a farm, fruit intake and family history of stomach cancer.	Multiplex PCR	7
Setiawan (2001) [38]	China	East Asians	73 histologically confirmed cases with gastric cancer	417 healthy cancer-free individuals	None.	Multiplex PCR	6
Saadat (2001) [36]	Iran	Caucasians	42 patients with pathologically confirmed primary gastric cancer	131 healthy blood donors matched with the patients according to age and gender	None.	Multiplex PCR	5
Setiawan (2000) [37]	China	East Asians	81 patients with pathologically confirmed diagnoses of gastric cancer	418 healthy and cancer-free individuals	Adjusted for sex, age, education, smoking, fruit intake, salt intake, H. pylori infection, and alcohol drinking.	Multiplex PCR	7
Katoh (1996) [21]	Japan	East Asians	139 patients with gastric cancer	126 subjects who had visited local medical clinics for regular medical health check-ups	None.	Multiplex PCR	5
Deakin (1996) [15]	UK	Caucasians	114 patients with gastric cancer	509 cancer-free individuals	None.	Multiplex PCR	5

(* PCR-CTPP, polymerase-chain-reaction with the confronting-two-pairprimer; Multiplex PCR, Multiplex polymerase-chain-reaction; Real-time PCR, Real-time polymerase-chain-reaction).

### Meta-analysis

**Figure 1:**
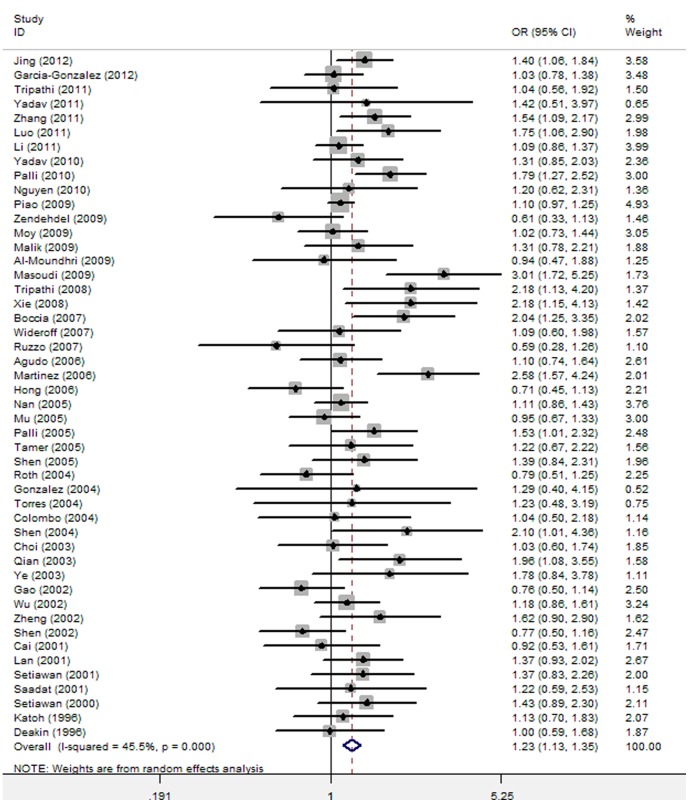
Meta-analysis of the association between *GSTT1* null genotype and gastric cancer risk. (48 studies, Random-effect model).

There was some heterogeneity among those 48 studies (I^2^ = 45.5%; P _Q statistic_ <0.001), thus the random-effect model (DerSimonian and Laird method) was used to pool the results (Table 2). Overall, *GSTT1* null genotype was significantly associated with increased risk of gastric cancer (Random-effect OR = 1.23, 95%CI 1.13–1.35, P _OR_ <0.001) (Figure 1, Table 2). In the subgroup analyses by ethnicity (Caucasians, East Asians, Africans, and Indians), there was an significant association between *GSTT1* null genotype and increased risk of gastric cancer in Caucasians (Random-effect OR = 1.30, 95%CI 1.06–1.59, P _OR_ = 0.010), East Asians (Random-effect OR = 1.16, 95%CI 1.05–1.29, P _OR_ = 0.003), and Indians (Fixed-effect OR = 1.37, 95%CI 1.06–1.77, P _OR_ = 0.017) (Table 2). In the subgroup analysis of studies with high quality, there was an obvious association between *GSTT1* null genotype and increased risk of gastric cancer (Random-effect OR = 1.23, 95%CI 1.12–1.35, P _OR_ <0.001) (Table 2).

**Table 2:** Meta-analysis of the association between *GSTT1* null genotype and gastric cancer risk.

Groups	Studies (Subjects)	OR (95%CI)	P _OR_	Pooled model	I^2^	P _Q statistic_
Total studies	48 (24,440)	1.23(1.13–1.35)	<0.001	Random-effect	45.5%	<0.001
Adjusted ORs	18 (8,339)	1.43(1.20–1.71)	<0.001	Random-effect	48.1%	0.012
Adjusted for H.pylori infection	5 (3,235)	1.70(1.43–2.01)	<0.001	Fixed-effect	18.5%	0.297
Caucasians	16 (8,178)	1.30(1.06–1.59)	0.010	Random-effect	61.4%	0.001
East Asians	25 (14,814)	1.16(1.05–1.29)	0.003	Random-effect	38.4%	0.028
Indians	5 (1,224)	1.37(1.06–1.77)	0.017	Fixed-effect	0.0%	0.590
Studies with high quality	43 (23,545)	1.23(1.12–1.35)	<0.001	Random-effect	49.2%	<0.001
Studies with low quality	5 (895)	1.31(0.95–1.80)	0.099	Fixed-effect	0.0%	0.513

(GSTT1, Glutathione S-transferase T1; 95%CI, 95% confidence interval; OR, odds ratio; P _OR,_ the P value of the pooled OR; P _Q statistic,_ the P value of the Q statistic).

**Figure 2:**
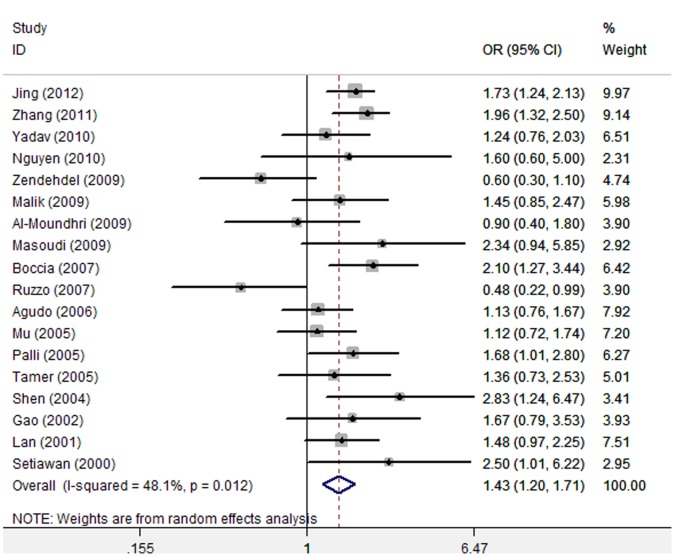
Assessment of the association between GSTT1 null genotype and gastric cancer risk by using adjusted estimates. (18 studies, Random-effect model).

After adjusting for other confounding variables, G*STT1* null genotype was still significantly associated with increased risk of gastric cancer (Random-effect OR = 1.43, 95%CI 1.20–1.71, P _OR_ <0.001, I^2^ = 48.1%) (Figure 2, Table 2). Meta-analysis of ORs adjusted for H.pylori infection also showed a significant association between *GSTT1* null genotype and increased risk of gastric cancer (OR = 1.34, 95%CI 1.09–1.64, P = 0.006) (Table 2).

### Publication Bias

**Figure 3:**
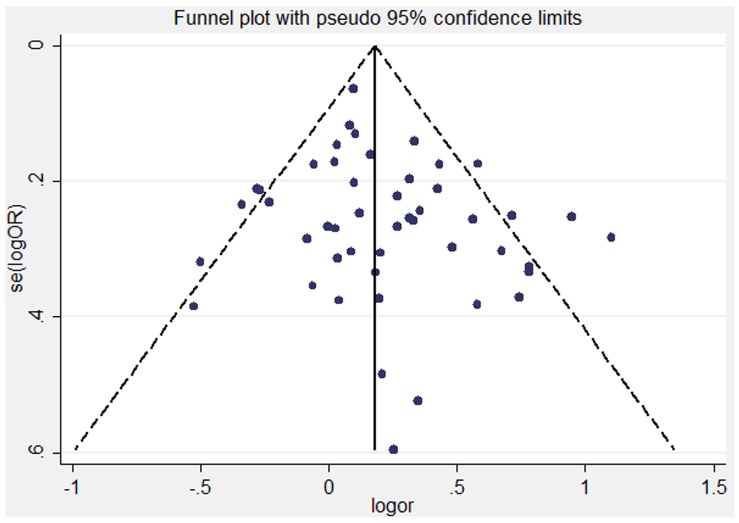
Funnel plots did not reveal any evidence of obvious asymmetry in the overall meta-analysis.

In the meta-analysis of total 48 studies, the shape of the funnel plot did not reveal any evidence of obvious asymmetry (Figure 3). In addition, both the Begg’s test and Egger’s test provided statistical evidence for the symmetry of the funnel plot (P _Begg_ = 0.333; P _Egger_ = 0.145). Therefore, there was no obvious risk of publication bias in the present meta-analysis.

## Discussion

Previous studies on the association between *GSTT1* polymorphism and gastric cancer risk reported inconclusive results. To clarify the possible association, we conducted a meta-analysis of a total of 48 studies with 24,440 individuals [9]–[48], [56]–[63]. Overall, *GSTT1* null genotype was significantly associated with increased risk of gastric cancer (Random-effect OR = 1.23, 95%CI 1.13–1.35, P _OR_ <0.001, I^2^ = 45.5%). Significant association was also found in Caucasians, East Asians, and Indians (P _Caucasians_ = 0.010; P _East Asians_ = 0.003; P _Indians_ = 0.017). After adjusting for other confounding variables, G*STT1* null genotype was also significantly associated with increased risk of gastric cancer (Random-effect OR = 1.43, 95%CI 1.20–1.71, P _OR_ <0.001, I^2^ = 48.1%). Therefore, the meta-analysis provides strong evidence for the significant association between *GSTT1* null genotype and increased risk of gastric cancer.

Endogenous products and environmental factors could result in the production of reactive oxygen species (ROS) and nitrogen metabolites causing cell injury and genetic instability [64], [65]. GSTs are the most important family of phase II isoenzymes known to detoxify a variety of electrophilic compounds, including carcinogens, chemotherapeutic drugs, environmental toxins, and DNA products generated by reactive oxygen species damage to intracellular molecules, chiefly by conjugating them with glutathione [66]. GSTs play a major role in cellular antimutagen and antioxidant defense mechanisms, and these enzymes may regulate pathways that prevent damage from several carcinogens. GSTs have proved to be involved in the detoxifying several carcinogens and may play an important role in carcinogenesis of cancer [66]. These enzymes also play a crucial role in protection of DNA from oxidative damage by ROS [66]. Therefore, the polymorphisms in *GSTT1* gene can causes the dysfunction of GSTs and result in less protection of DNA from damages caused by ROS [8]. The null genotype of *GSTT1* gene can cause the complete absence of GST enzymes activity, which may increase the host’s susceptibility to DNA damage and some cancers. Thus, there is obvious biochemical evidence for the relationship of *GSTT1* polymorphism with cancer risk [8].

Nowadays, a great number of studies have been published to assess the association between *GSTT1* null genotype and risks of some cancers. Currently, *GSTT1* null genotype has been proven to be associated with risks of some cancers, such as lung cancer and hepatocellular carcinoma [67], [68]. The significant associations further suggest that *GSTT1* null genotype can affect the individual susceptibility to common malignancies, and has important roles the carcinogenesis of some cancers.

A meta-analysis in 2010 was performed to assess the association between GSTT1 null genotype and risk of gastric cancer by including thirty-six studies with 4,357 gastric cancer cases and 9,796 controls [69]. The previous meta-analysis concluded that GSTT1 gene polymorphism may be not associated with increased gastric cancer risk among Europeans, Americans, and East Asians, and more large-scale studies based on the same racial group were needed [69]. In the present meta-analysis, we performed a updated literature search and included 12 new studies, and the total sample size (24, 440 individuals) was nearly two times of that from the previous meta-analysis. To the best our knowledge, our meta-analysis is the largest meta-analysis of the association between *GSTT1* null genotype and gastric cancer risk. Therefore, compared with the previous meta-analysis, the present meta-analysis has greater statistical power and can provide a more precise assessment on the association between *GSTT1* null genotype and gastric cancer risk.

Some limitations of this study should be acknowledged. Firstly, there was some heterogeneity in both the meta-analysis of total 48 studies and the subgroup analyses by ethnicity. The differences from the selection criteria of cases or controls, the adjusted confounding variables, and the ethnicity result in the heterogeneity. Secondly, most studies in the meta-analysis were retrospective design which could suffer more risk of bias owing to the methodological deficiency of retrospective studies. Those there was no obvious risk of publication bias in the present meta-analysis, the risks of other potential bias were unable to be excluded. Some misclassification bias was possible because most studies could not exclude latent gastric cancer cases in the control group. Therefore, more studies with prospective design and low risk of other bias are needed to provide a more precise estimate of the association between *GSTT1* null genotype and gastric cancer risk. Finally, we could not address gene-gene and gene-environmental interactions in the association between *GSTT1* null genotype and gastric cancer risk. The latter may be important for genes that code proteins with detoxifying function, but would require detailed information on exposures to various potential carcinogens and individual-level data and would be most meaningful only for common exposures that are found to be strong risk factors for the disease. Thus, more studies analyses on the gene-gene and gene-environmental interactions are needed.

In conclusion, the meta-analysis provides strong evidence for the significant association between *GSTT1* null genotype and increased risk of gastric cancer. In addition, more studies with well design are needed to further assess the possible gene-gene and gene-environmental interactions in the association between *GSTT1* null genotype and gastric cancer risk.

## Supporting Information

Figure S1 **Flow diagram in the meta-analysis of the association between GSTT1 null genotype and gastric cancer risk.**
(TIF)
