# Supplementary figures and images for: Association between Glutathione S-Transferase T1 Null Genotype and Gastric Cancer Risk: A Meta-Analysis of 48 Studies
Source: PLoS One. 2013 Apr 9;8(4):e60833. doi: 10.1371/journal.pone.0060833 (PMC3621870; doi:10.1371/journal.pone.0060833)

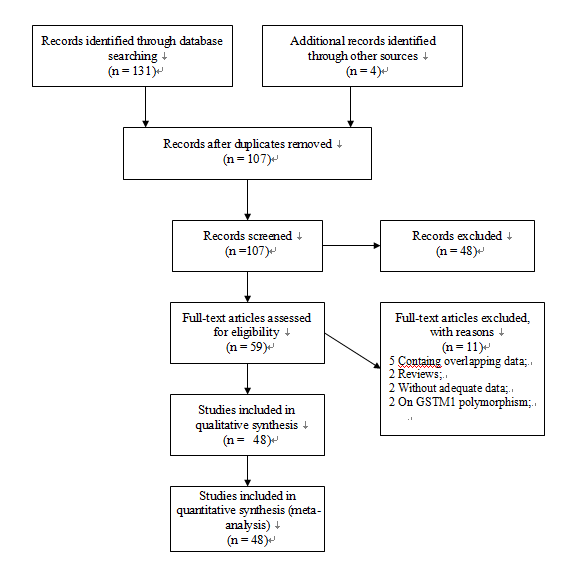

Supplement: Figure S1 — Flow diagram in the meta-analysis of the association between GSTT1 null genotype and gastric cancer risk. (TIF) [file pone.0060833.s001.tif]
